# Spinning Babies^®^ approach: A way to promote fetal head rotation during labor?

**DOI:** 10.18332/ejm/206972

**Published:** 2025-09-05

**Authors:** Marta Mazzeo Melchionda, Tamara Aloi, Francesca Bruno, Alessia De Lazzari, Paola A. Mauri, Giovanna Esposito

**Affiliations:** 1 Midwifery School, Department of Clinical Sciences and Community Health, Dipartimento di Eccellenza 2023-2027, University of Milan, Milan, Italy; 2 Department of Woman, Newborn and Child, Fondazione Istituto di Ricovero e Cura a Carattere Scientifico Ca’ Granda Ospedale Maggiore Policlinico, Milan, Italy; 3 Obstetrics and Gynecology Clinic, Macedonio Melloni Hospital, Milan, Italy

**Keywords:** occiput posterior position, occiput transverse position, Spinning Babies^®^

## Abstract

**INTRODUCTION:**

Optimal fetal positioning is essential for ensuring that labor progresses efficiently and reducing the need for interventions. The aim of this study was to evaluate the potential role of the Spinning Babies^®^ approach to facilitate fetal head rotation.

**METHODS:**

This retrospective study was based on data collected by midwifery students, supervised by experienced midwifery tutors, using digital partograms. The fetal position was recorded at several stages of labor, particularly at the labor onset and at the delivery. For the current analysis, only women with an occiput-posterior or occiput-transverse fetal position were included. The Spinning Babies^®^ approach was compared with a control group. The association between anterior head rotation and the use of the Spinning Babies^®^ techniques was evaluated using a log-binomial model.

**RESULTS:**

A total of 244 partograms were identified. Of these, 60 women underwent the Spinning Babies^®^ approach, while the remaining 184 did not (control group). At first, in the Spinning Babies^®^ group, 40.0% were left occiput-posterior, 43.3% were right occiput-posterior, and 16.7% were occiput-transverse. In the control group the corresponding proportions were: 33.2%, 56.0%, and 10.9%. At the delivery, the fetal head rotated from posterior (or transverse) to anterior in 93.3% of women who underwent Spinning Babies^®^ techniques, compared to 63.6% of controls. The use of Spinning Babies^®^ techniques was associated with a 45% increased likelihood of achieving anterior position (RR=1.45; 95% CI: 1.23–1.72).

**CONCLUSIONS:**

Optimal fetal positioning may be facilitated using Spinning Babies^®^ approach. To confirm these findings, further research using more standardized protocols and objective measures is needed.

## INTRODUCTION

The biomechanics of childbirth involve the complex interplay between the structure of the mother’s pelvis, the position and progress of the fetus in the birth canal, and uterine contractions. The fetus may be presented in a position that is a deviation from the optimal orientation for the delivery, where the fetus’s head is down and facing the mother’s spine. The occiput-posterior position is the most common fetal non-optimal position that occurs during labor, approximately 0.2–8% of patients deliver in this position^1^. However, many non-optimal positions (>80%) identified at the onset of labor resolve as the fetus rotates into a more favorable position during the progression of labor^2^.

Spinning Babies^®^ is a physiological approach^3^ developed by American midwife Gail Tully. It includes several exercises and techniques aimed to optimize fetal positioning throughout pregnancy and labor, which require expert guidance. It is based on three principles: balance, gravity, and movement. Balance involves maintaining optimal tension in the muscles and ligaments of the uterus to facilitate the fetus’s natural progress through the birth canal and the adjustment of head position. In cases where the baby is poorly positioned due to tight pelvic muscles, softening these muscles and ligaments can increase pelvic flexibility and help the descending. Only once balance is achieved, gravity and movement come into play and can be effective. Using gravity through maternal positioning and staying active helps to reduce pain and encourages the baby to descend lower. Being upright and mobile during labor is often more manageable than lying on hips or back in bed. Increased pelvic mobility can make it more likely that the baby will turn.

At our university teaching unit in Milan, northern Italy, the Spinning Babies^®^ approach was proposed to a sample of women diagnosed with a fetal non-optimal position. The aim of the current study is to compare the rate of anterior head rotation between this group and a control group which did not undergo the techniques.

## METHODS

### Data sources and population study

This was a retrospective study based on data from digital partograms of deliveries in which the fetus was in an occiput-posterior or occiput-transverse position at the onset of labor. The partograms, including information on maternal characteristics, current and remote obstetric history, details of labor progress and birth outcomes, were systematically collected by midwifery students under the supervision of experienced midwifery tutors at the Fondazione IRCCS Ca’ Granda Ospedale Maggiore Policlinico of Milan and other centers associated with the School of Midwifery between 2016 and 2024, during randomly selected periods of the year according to the training period.

A total of 1931 partograms for which information on fetal position was available at the onset of labor and at the delivery were identified. Out of these, 244 partograms of deliveries with fetus in an occiput-posterior or occiput-transverse position at the onset of labor were selected. We did not include cases that ended with a cesarean section, because the fetal position at delivery was not available for these cases.

### Fetal position diagnosis and Spinning Babies^®^ approach

The fetal position was routinely recorded at multiple stages during labor, specifically at the onset of labor and at delivery. This was determined through vaginal examinations conducted by the attending midwife. This systematic approach enabled the accurate identification of the fetal position and rotation at critical stages of labor, thereby facilitating the optimal management of the delivery process. In the last two years, the Spinning Babies^®^ approach has been introduced and offered to women in cases of fetal non-optimal position. For those who accepted it and underwent the approach, this was recorded and described in the partogram.

### Data analysis

Categorical data were presented as absolute and relative frequencies (percentages), and continuous data as means and standard deviations. The distribution of the baseline characteristics was compared using Fisher’s exact test for categorical variables, while continuous variables were compared using t-test for independent samples.

The proportions of fetuses that rotated and assumed an anterior position were calculated for both the Spinning Babies^®^ group and the control group. The association between anterior position and the use of Spinning Babies^®^ techniques was evaluated using a log-binomial model, accounting for parity and analgesia. The risk ratio (RR) and 95% confidence interval (CI) is provided.

The study was carried out in accordance with the ethical standards for research involving human subjects as laid down in the Declaration of Helsinki. The study protocol was approved by the Ethical Review Board of Fondazione IRCCS Ca’ Granda Ospedale Maggiore Policlinico, Milano, Italy.

## RESULTS

Of the 244 deliveries identified, 60 women underwent the Spinning Babies^®^ approach (mean age: 33.5 ± 4.7 years) and 184 women did not (mean age: 32.4 ± 5.1 years).

Women who underwent the Spinning Babies^®^ approach were more likely to be nulliparous (83.3% vs 69.0%, p=0.03) and to use analgesia (90.0% vs 62.5%, p<0.01). No differences emerged for use of oxytocin (55.7% vs 43.3%, p=0.09).

The distribution of fetal position at onset of labor and at delivery in the two groups is reported in Table 1. At the delivery, fetal head rotated from posterior (or transverse) to anterior in 93.3% (56/60) of cases of women who underwent Spinning Babies^®^ approach and 63.6% (117/184) of controls. The use of Spinning Babies^®^ techniques was associated with a 45% increased likelihood of achieving anterior positioning, with a RR of 1.45 (95% CI: 1.23–1.72), after adjusting for parity and analgesia.

**Table 1:** Fetal position at onset of labor and at delivery in the Spinning Babies^®^ and control groups

*Fetal position*	*Spinning Babies^®^ approach*		*Controls*	
	*n*	*%*	*n*	*%*
**At onset of labor**				
Left occiput-posterior	24	40.0	61	33.2
Right occiput-posterior	26	43.3	103	56.0
Occiput-transverse	10	16.7	20	10.9
**At delivery**				
Occiput-anterior	56	93.3	117	63.6
Left occiput-posterior	2	3.3	22	12.0
Right occiput-posterior	2	3.3	37	20.1
Occiput-transverse	0	0.0	8	4.3

## DISCUSSION

The Spinning Babies^®^ approach appears to facilitate fetal rotation, thereby achieving optimal positioning during labor. In our sample, the majority of women who underwent these techniques achieved optimal fetal positioning by rotation (>90%), whereas approximately 65% of those who did not perform the techniques achieved fetal rotation.

Our findings were consistent with previous evidence that found a potential benefit of a set of interventions combining Spinning Babies^®^ approach and the Rebozo technique in reducing the likelihood of persistent occiput-posterior position^4^. Further, the literature suggests that alternative maternal positions may have a positive effect on labor, reducing maternal pain, operative vaginal delivery, cesarean section, and episiotomy rates. Women should be encouraged to move and give birth in the most comfortable position^5^.

The modified Sims position as a maternal postural intervention has been found to be effective in facilitating fetal rotation, leading to a reduction in cesarean delivery rates^6^. In a study of women who did not receive epidural anesthesia, both the semi-prone and knee-chest positions were associated with increased spontaneous rotation of the occiput to the anterior position, higher rates of vaginal delivery, and a reduction in the duration of the active phase of labor, as well as reduced post-delivery back pain^7^. However, there are conflicting results, as other authors have failed to demonstrate a significant benefit of the hands and knees position in correcting the occiput-posterior position of the fetus during the first stage of labor; however, women in the study reported an increase in comfort^8^. A systematic review and meta-analysis found that the majority of fetuses spontaneously rotate to an occiput-anterior position and suggested that maternal posture may facilitate earlier rotation but has no effect on the subset of infants who would otherwise remain in the occiput-posterior position until birth^9^. Also, the most recent Cochrane review noted a lack of evidence to guide practice regarding positional interventions for fetal non-optimal position in late pregnancy^10^.

In addition, a state of dynamic equilibrium within the uterine and pelvic structures must be actively maintained, highlighting the crucial role of the principle of balance^3^. This principle is shared by other disciplines such as osteopathy, a complementary and manual approach that uses various techniques, including myofascial techniques, to increase range of motion, improve tissue texture, reduce pain, and restore symmetry^11^.

The head of the fetus encounters a number of obstacles as it descends, flexes and rotates in the birth canal. The position it assumes reflects the structural conformation of the mother, determined not only by the measurement of the pelvic diameters defined by the bony boundaries, but also by the tensions or poor tone of the soft tissues. From this perspective, antenatal preparation of the pelvic floor through massage^12^ and training^13^ is paramount. The pelvic floor training before birth has been associated with notably lower rates of episiotomies and severe perineal trauma, as well as a higher probability of an intact perineum compared to women who received only standard care^14^.

Another target structure of the Spinning Babies^®^ approach, as of other manual approaches, is the fascia, a connective tissue that suspends, protects, and connects various structures creating a seamless connection within the human body. The fascia reorganizes itself along the lines of tension imposed or expressed in the body, in a way that can affect fascial restrictions throughout the body. This can potentially stress all the structures, both near and far, that are enveloped by the fascia itself, with consequent mechanical and physiological effects that cause misalignment with the typical position^15^. As regards specific techniques, the abdominal release proposed in the Spinning Babies^®^ program uses a very light touch to release fascial system around the pelvis and respiratory diaphragms, rebalancing the tough fascia that wraps around the sacrum. This is likely to allow the lower uterine segment to relax from tensioning the sacrum. The release of the diaphragm also helps to relax the broad ligament, which gives the baby more room to get into the right position for the birth.

### Limitations

This study has limitations. Firstly, the diagnosis of the fetal position was made through digital examination rather than systematic ultrasound confirmation, which may have introduced variability or misclassification. Secondly, the Spinning Babies^®^ techniques were applied inconsistently in terms of timing, sequence and frequency, which may have affected their effectiveness. Not all women underwent the same combination of techniques, and the order in which they were applied varied between cases and women were not randomized and participation in the Spinning Babies^®^ approach was voluntary. Additionally, although we adjusted for relevant variables such as parity and analgesia use, residual confounding remains possible due to unmeasured factors, including provider experience. These factors may have influenced both fetal rotation and the decision to apply, or the effectiveness of, the techniques. Finally, excluding cases ending in cesarean section due to the inability to determine the fetal position at delivery may have introduced selection bias, limiting the generalizability of the findings.

## CONCLUSIONS

This study suggests that the use of Spinning Babies^®^ approach may favor optimal fetal positioning. Further prospective studies employing standardized methodologies and objective evaluation tools are needed to confirm and expand upon these results.

## Data Availability

The data supporting this research are available from the authors on reasonable request.
